# Isolation and Characterization of a Novel Electrogenic Bacterium, *Dietzia* sp. RNV-4

**DOI:** 10.1371/journal.pone.0169955

**Published:** 2017-02-13

**Authors:** Natalia J. Sacco, M. Celina Bonetto, Eduardo Cortón

**Affiliations:** Laboratory of Biosensors and Bioanalysis (LABB), Departamento de Química Biológica and IQUIBICEN-CONICET, Facultad de Ciencias Exactas y Naturales, Universidad de Buenos Aires, Ciudad Universitaria, Ciudad Autónoma de Buenos Aires, Buenos Aires, Argentina; National Renewable Energy Laboratory, UNITED STATES

## Abstract

Electrogenic bacteria are organisms that can transfer electrons to extracellular electron acceptors and have the potential to be used in devices such as bioelectrochemical systems (BES). In this study, *Dietzia* sp. RNV-4 bacterium has been isolated and identified based on its biochemical, physiological and morphological characteristics, as well as by its 16S rRNA sequence analysis. Furthermore, the current density production and electron transfer mechanisms were investigated using bioelectrochemical methods. The chronoamperometric data showed that the biofilm of *Dietzia* sp. RNV-4 grew as the current increased with time, reaching a maximum of 176.6 ± 66.1 mA/m^2^ at the end of the experiment (7 d); this highly suggests that the current was generated by the biofilm. The main electron transfer mechanism, indicated by the cyclic voltammograms, was due to secreted redox mediators. By high performance liquid chromatography, canthaxanthin was identified as the main compound involved in charge transfer between the bacteria and the solid electrodes. *Dietzia* sp. RNV-4 was used as biological material in a microbial fuel cell (MFC) and the current density production was 299.4 ± 40.2 mA/m^2^. This is the first time that *Dietzia* sp. RNV-4 has been electrochemically characterized and identified as a new electrogenic strain.

## Introduction

Electrogenic microorganisms are a very heterogeneous group of organisms, not defined by taxonomical, physiological or other relevant biological characteristics. The name is just a useful way to describe those organisms which are in some way able to transfer electrical charge from or to a solid electrode [1]. Some discussion exists in the discipline, and there is a blurry line among electrogenic and non-electrogenic organisms. Nevertheless, bacteria of the genus *Geobacter* and *Shewanella* are considered promising electron generators for microbial fuel cells (MFCs), and because of that, they are the most studied genera. But some limitations for the practical applications in industrial or research are commented below.

*G*. *sulfurreducens* is one of most extensively studied microorganisms capable of high current densities in a MFC. This organism has become a model for bacterial processes in a MFC since: it is representative of *Geobacter* species commonly enriched electrodes (anodes), when environmental samples are used to inoculate a MFC [2]; also pure cultures of *G*. *sulfurreducens* have been found to produce near or greater than maximum power of mixed species biofilms [3]. Furthermore, *G sulfurreducens* belongs to a class of microbes referred to as electrogenic, a term used to describe microbes that conserve energy to support growth by completely oxidizing organic compounds to carbon dioxide with direct electron transfer to the anode of the MFC. *Geobacter* species have been shown to be important in the anaerobic degradation of different (but limited) carbon sources. Due to the extreme intolerance of most *Geobacter* species to oxygen, technological and research possibilities are limited.

*Shewanella oneidensi*s, on the other hand, is able to survive in presence of oxygen, but an important disadvantage is that it did not completely oxidize the organic substrate typically used (lactate) in a MFC, leaving electrons unutilized and waste products such as acetate. The columbic efficiency shown was about 56.2%. The spectrum of carbon substrates that this bacterium can use is limited. When microorganisms thriving in a MFC system is capable of completely oxidize the organic substrates to CO_2_ higher columbic efficiencies have been reported [4].

But the search of other organisms with electrogenic capabilities as well of other positive/diverse characteristics to be used in MFCs, are needed in order to truly explore the possibilities of this systems as electricity sources, wastewater treatment systems or as part of biosensors and bioassays [1].

MFCs are BES typically composed of two compartments separated by a cation-exchange membrane (as Nafion); more recently, single chamber MFCs have been proposed (air-cathode MFCs), or systems where no membranes (but physical separation) exists between anode and cathode (as in sedimentary MFCs). In an archetypal set-up, bacteria oxidize organic matter and transfer electrons to the anode, at the same time protons are liberated in the anodic compartment. The electrons can flow to the cathode through an external conductor, whereas protons are able to travel across the ion-exchange membrane. At the cathode, electrons and protons combine to oxygen to form water [1,5].

Researchers have proposed three distinct extracellular electron transfer (EET) mechanisms for electron transfer to solid electrodes [6]. The first EET mechanism proposes the presence of a soluble electron mediator or shuttle, typically a low molecular weight organic molecule that has the ability to participate in redox reactions. Mediators should be ideally chemically stable and not easily biodegraded. Bacteria can use either exogenous or endogenous (produced by bacteria) shuttle compounds for extracellular electron transport. They can diffuse in and out through the bacterial cell trapping and transporting electrons. Reduced soluble shuttles can diffuse out of the cell and into the medium and are then able to pass on the electrons to suitable external acceptors, as insoluble Fe (III) oxides, or an MFC anode [7]. Some bacteria are known to produce their own electron shuttles compounds such as melanin, quinones, phenazines, riboflavin and flavin mononucleotide [8–11]. A second proposed EET mechanism is the direct electron transfer from bacteria to the electrode. The presence of outer-membrane cytochromes allows this cell/electrode interaction directly. The third mechanism proposed is the formation of an extracellular biofilm matrix with redox or conductive capacity, including extracellular DNA, RNA, cytochromes or other relatively non-mobile molecules, capable to help the electron transfer from or to the bacteria in the proximity of the electrode [12]. An example of a new possible mechanism was made by Reguera and colleagues [13], who proposed that cellular pili can act as conductive nanowires [14].

Most investigations related to the EET in bacteria have been focused on two relevant microorganisms, *Geobacter sulfurreducens* [15,16] and *Shewanella oneidensis* [17–19] and showed that specific genes and proteins were involved in these processes. *S*. *oneidensis* interacts with the electrodes primarily using flavins, actively secreted by the cells, as soluble electron shuttles [9]. *Geobacter sulfurreducens* makes direct electrical contacts with electrodes via cytochromes c-type, present in the bacterial membrane surface facing outside [12,20,21]. Further studies of the electroactive bacteria (EAB) and biofilms will benefit from the isolation and identification of other microorganisms able to transfer electrons to an electrode. The microbial community or the specific microorganisms on the anode are now becoming relevant factors in power production of the MFCs [22–24]. Moreover, new microorganisms with their particular physiology and metabolisms can be used as the starting material to develop new bioassays and biosensors [1,25]. Therefore, it is important to isolate and understand the physiology of new EAB and the ecology of the communities on the electrodes [26].

Our main objective is to find and characterize a new and versatile electrogenic strain, to be used as biological material in MFC and biosensors. In this work, we were able to isolate a bacterium which was identified as *Dietzia* sp. which we call *Dietzia* sp. RNV-4. The biofilm forming capacity, current density production and electron transfer mechanisms of *Dietzia* sp. RNV-4 growing over Toray paper carbon electrodes were investigated in potentiostat-controlled electrochemical cells, poised at 0.24 V (vs. Ag/AgCl). The results show that *Dietzia* sp. RNV-4 is a good candidate as an electrogenic microorganism, presenting an EET mechanism to solid electrodes using canthaxanthin as electron shuttle. Several relevant characteristics for applications such as power production or as a part of biosensors systems will be discussed here.

## Materials and Methods

### Construction of the sedimentary microbial fuel cell, SMFC

SMFC were assembled as described by Sacco *et al*. [27]. Briefly, the sediment was collected from the shore of the Río de la Plata River on the intertidal zone (maintaining the soil structure). Anoxic sediments were used to fill up 1 L beakers up to 3/4 of its total volume. The anodes were completely embedded horizontally in the sediment at a distance of 7 cm below the surface, while the cathodes were suspended (also horizontally) in the overlying freshwater at a distance of 5 cm from the sediment surface. The overlying water was taken from the sampling sites, and was continuously bubbled with air using an aquarium air-pump in order to maintain saturated oxygen conditions. Water lost by evaporation was replaced with double osmosis water. Sediments were equilibrated under open circuit condition for 48 h. Afterwards, the anode was connected to the cathode via a fixed external load of 4.6 kΩ. All SMFCs were operated at room temperature (25°C). Plain graphite electrodes were used as cathode and anode.

### Microbiological techniques used for isolation and identification of the RNV-4 strain

In order to isolate the microorganisms growing over the anodes, the surface of the electrodes were rinsed with a stream of sterile double osmosis water until they were free of visible debris. Approximately the first millimeter of the graphite electrode (the anode, which was in the mud) was scraped vigorously with a sterile razor blade into 1.5 mL phosphate buffer, 50 mM, pH 7.2, producing a suspension consisting of graphite and electrode-associated microbes. The obtained suspension was serially diluted up to 10^−6^ (graphite/cell dilutions). 20 mL serum vials were stoppered with butyl rubber bungs, clamped with aluminum caps containing 10 mL of the appropriate medium, and inoculated with 0.25 mL of each graphite/cell dilution. Tubes from the highest dilutions showing growth after 15 days were used to inoculate new dilutions with the same medium.

Cultures were, in this way, three times diluted to extinction. The medium contained (in g/L): NaHCO_3_ (2.5), NH_4_Cl (0.5), yeast extract (0.2), peptone (0.1), NaH_2_PO_4_.H_2_O (0.6), KCl (0.1), ferric citrate (13.7), sodium acetate (6.8) and lactic acid (1.5), in distilled water and adjusted to pH 7.2. For solid medium plates, agar (15) was added. These media were autoclaved for sterilization.

Microorganisms were also isolated from the same sediment sample used to start the SMFCs; 1 g of sediment was suspended in 9 mL of sterile physiological solution and mixed by vortexing for 10 min. The sediment was decanted and 1 mL of the supernatant solution was serially diluted up to 10^−6^ and used as the graphite/cell dilution abovementioned. In both isolation protocols, from anodes and mud, *Dietzia* sp. RNV-4 was isolated.

*Dietzia* sp. RNV-4 was grown and maintained on Brain-Heart Infusion (BHI) Agar, containing (in g/L): brain heart infusion (solids) (8), dextrose (2), peptic digest of animal tissue (5), Na_2_HPO_4_ (2.5), pancreatic digest of casein (16), NaCl (5), agar (15). Final pH: 7.4 ± 0.2. (DIFCO, Becton, Dickinson and Company, Maryland, US). One colony from a *Dietzia* sp. RNV-4 BHI culture plate was inoculated in BHI liquid media (without agar) and cultured for 72 h at 32°C when biomass was needed.

In experiments where strictly anaerobic techniques were employed, 20 mL of anaerobic medium was dispensed into serum vials, then inoculated with 0.25 mL of each graphite/cell dilution, bubbled with sterile N_2_ for 10 min, and sealed immediately afterwards with butyl rubber stoppers and crimped with aluminum caps [28].

The strains tested for anaerobic growth were streaked to obtain single colonies and the plates were incubated in an anaerobic jar (GasPak; Becton, Dickinson and Company, Maryland, US). All media were autoclaved for sterilization.

The extraction of nucleic acids, the PCR amplification, and the 16S rRNA sequencing process were performed by Macrogen Inc. (Seoul, South Korea). Universal primers (518F: 5’-CCAGCAGCCGCGGTAATACG-3’ and 800R: 5’-ACCAGGGTATCTAATCC-3’) were used for PCR amplification and sequencing processes, and the nucleotide sequence data were obtained using the Big Dye^™^ terminator cycle sequencing kit (Applied Biosystems, Foster City, CA, USA) and the ABI 3730 XL analyzer (Applied Biosystems).

The sequence obtained was compared with the sequences of reference species of bacteria contained in the genomic database banks, using the BLAST sequence alignment tool (https://blast.ncbi.nlm.nih.gov/Blast.cgi?PROGRAM=blastn&PAGE_TYPE=BlastSearch&LINK_LOC=blasthome).

### Bioinformatic and phylogenetic analyses

Genome sequence analysis of RNV-4 strain was analyzed using the following program available on line: BLAST (http://blast.ncbi.nlm.nih.gov/Blast.cgi). Phylogenetic and molecular evolutionary analyses were conducted using MEGA version 7 [29]. Phylogenetic trees were constructed using the neighbor-joining method with genetic distances computed using Poisson correction model and maximum parsimony method, bootstrap analysis of 500 replicates and root on midpoint.

### Morphological characterization

Gram’s staining was performed as the procedures described in Merchant and Packer [30] to determine the size, shape and arrangement of bacteria. Spore formation was observed according to Schaeffer-Fulton [31] method for staining endospores. A microscope (Eclipse E600, Nikon, Nikon Instech Co., Ltd., Karagawa, Japan), was used to all microscopic studies.

### Physiological and biochemical properties

The biofilm forming capacity was examined according to the method described by Christensen [32]. This test is the most widely used and it is considered as a standard test for detection of biofilm formation. In the present study, given the slow growth rate of the bacterium used, the incubation was extended to 72 h.

One colony from a RNV-4 BHI agar plate was inoculated in BHI liquid media and cultured for 72 h at 32°C in a stationary condition. 1 mL of this culture was inoculated into glass tubes (making a 1/5 dilution of the original culture) and was incubated for 96 h at 32°C in both, aerobic and anaerobic (bubbling with sterile N_2_) condition.

After the incubation, the content of each glass tube was gently removed, and washed four times with 10 mL of sterile saline solution (NaCl 0.9% w/v) to remove free-floating “planktonic” bacteria. Biofilms formed by adherent “sessile” organisms in tube were first fixed with acetic acid (30% w/v), immediately afterwards with ethanol (96% v/v), and finally stained with crystal violet (0.1% w/v) solutions. Excess of stain was rinsed off by thoroughly washing with deionized water and later the tubes were kept for drying. The test was considered positive when there was an adherent layer of stained material on the inner surface of the tube (n = 3). Optical densities of stained adherent bacteria were determined with an UV-Visible spectrophotometer, UV-160A (Shimadzu, U.S.) at wavelength of 570 nm (OD_570_). These OD values were considered as an index of bacteria adhering to surface and forming biofilm. To compensate for background absorbance, OD readings from sterile medium, fixative and dye were averaged and subtracted from all test values.

To differentiate the oxygen requirement level of the RNV-4 strain, we used fluid thioglycollate medium (Laboratorios Britania S.A, Argentina). The catalase test is also valuable in differentiating aerobic and obligate anaerobic bacteria, as anaerobes are generally known to lack this enzyme. So, we performed the classical catalase test described by Mahon [33], where a small amount of bacterial colony was transferred to a clean surface of a dry glass slide using a loop, and then a drop of 3% H_2_O_2_ was placed onto the slide and mixed. The rapid evolution of oxygen (within 5–10 s) as evidenced by bubbling was a positive result.

We used a modified Gaby and Hadley oxidase test [34], soaking a small piece of filter paper in oxidase reagent (1% w/v *p*-aminodimethylaniline oxalate, DIFCO, in distilled water) and left to dry. A colony from a fresh bacterial plate was picked with a sterile tip and rubbed on the treated filter paper. We observed the color changes afterwards.

### Bacteria and growth medium

One colony from a *Dietzia* sp. RNV-4 growing at a BHI (an enriched non-specific medium) agar plate was inoculated in liquid basal medium (BM) described by Baron as SBM media [20], and grown aerobically at 32°C for 72 h, under shaking conditions at 100 rpm. *S*. *oneidensis* MR-1 from a frozen stock was grown aerobically in 100 mL of Luria-Bertani (LB) media, at 32°C for 24 h, under shaking conditions at 100 rpm.

### Bioelectrochemical system BES setup

**Fig 1:**
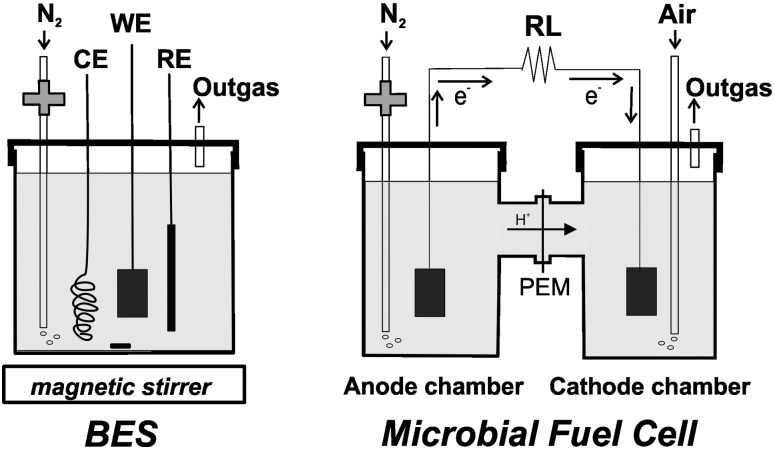
Schematic diagram of the bioelectrochemical set-up used in this work. In the denominated bioelectrochemical system (BES) a working electrode (WE) is covered by a biofilm; CE and RE are immersed in the same solution (left). In the microbial fuel cell (MFC) the anode and cathode compartments were separated by a PEM (Nafion) membrane (right). N_2_ was filtered by a 0.22 μm membrane, as shown.

A 100 mL bottle (Duran^®^, Germany) with a screw cap containing 4 ports, with threaded joints and silicone seals each, was used. One of the ports was used for N_2_ bubbling, placing a 1.6 mm diameter and 20 cm long Teflon hose, with a 0.22 μm Teflon membrane filter at the upper end to avoid contamination. The WE, RE and CE were placed in each of the three remaining ports (Fig 1).

### Electrode preparation to BES

Carbon paper TGP-H-030 (Toray^®^), with a density of 0.40 g/cm^3^and a porosity of 80%, was cut into 1 x 1.5 cm rectangles and used as working electrodes (WE) after connecting each of them to an insulated copper electrical wire. Lab-made saturated Ag/AgCl reference electrodes (RE) were used, preparing a new one before each assay. A coil made of 1 mm diameter of a stainless steel wire, NiCr (DIN 1.4310), was used as a counter electrode (CE). WEs were exposed to a 1 M of HCl and a 1 M of NaOH solutions (1 h in each solution) for cleaning after each measurement, and stored in sterile distilled water.

### Assembly and operation of the BES

25 mL of a *Dietzia* sp. RNV-4 liquid culture, with an optical density of 1.0 ± 0.1 at a λ = 610 nm (OD_610_) were transferred to the BES containing 25 mL of BM, a magnetic bar, and 20 mM lactate. Afterwards, the BES was purged for 50 min with humidified sterile oxygen-free N_2_ and placed onto a magnetic stirrer. The BES was maintained at 32°C and the electrodes were connected to a Potentiostat/Galvanostat/ZRA (Series G 300^™^ from Gamry Instruments Inc. Warminster, US). After 48 h, the spent growth medium was replaced with fresh BM with 20 mM of lactate, to promote the EAB growth. Following the first medium change (MC), 20 mM of lactate were injected each time the current decreased, to maintain a non-limiting electron donor concentration in the BES. At the end of the experiment (187 h), the BES media (50 mL) was removed and centrifuged (1842 *g* for 15 min), and the bacterial pellet obtained was resuspended in 50 mL of BM with 20 mM of lactate (bacterial suspension). Both, the cell-free supernatant and the bacterial suspension were studied by cyclic voltammetry (CV).

### Extraction and measurement of carotenoids

The extraction of total carotenoids from culture supernatants was made as described in Esfahani-Mashhour [35] with a modification, methanol:acetone in proportion 6:4 was used as extraction solvent mixture. We used a UV-Visible spectrophotometer (UV-160A, Shimadzu, U.S.) to scan the samples.

CanthaPlus 10% (Novepha, China), containing about 10% of canthaxanthin crystals (IUPAC name: β,β-Carotene-4,4'-dione), 1.5% ethoxyquin, 1% ascorbylpalmitate, 10% gelatin, 60% cornstarch, 5.5% sucrose and 12% dextrin was used as comparison, and canthaxanthin carotenoid was extracted also using the Esfahani-Mashhour modified protocol mentioned in the previous paragraph, and denominated commercial canthaxanthin extract. Both extracts were compared.

An aliquot from each extract was taken, and processed for HPLC analysis for identification of the carotenoid/s. Briefly, carotenoid analysis was performed on a Spectra Physics HPLC system equipped with a variable wavelength detector (Spectra Series UV100) measuring absorbance at 470 nm, with an integrator Data Jet SP4600, the stationary phase was composed of a C18 reversed-phase column, (3.9 mm X 300 mm, μBondapack, Waters Corp, Milford, US). The mobile phase was methanol 0.5 mL/min, chart speed of 0.5 cm/min.

### MFC construction and operation

The MFC consisted of an anode and a cathode placed on opposite sides in a plastic bottle with a volume of 100 mL, joined together on either side of a proton exchange membrane (Nafion 117, 1 cm^2^) (Fig 1). The membrane was pretreated by boiling in H_2_O_2_ (3%) and deionized water followed by 3% H_2_SO_4_ and deionized water, each for 2 h, and then stored in water prior to being used. The anode and cathode were made of carbon (Toray) paper. The cathode chamber contained phosphate buffer 100 mM, pH 7.0; NaCl (4.5 g/L) and ferricyanide (8.4 g/L). The anode compartment was inoculated with a 10:90 mixture of inoculum with *Dietzia* and medium BM, previously was gassed 50 min with humidified sterile oxygen-free N_2_. To maintain the aerobic condition of the cathode compartment, air was gassed at the same time.

Potential (E) was daily measured by a multimeter with a data acquisition system (UNI-T, Uni-Trend Technology, China). Current (i) was calculated as i = E/R, where R was the external circuit resistor (R_L_, load resistor), that could be easily replaced. Power (P) was calculated as P = iE. The power density (P_D_) and current density (j) values were the P and i values normalized by the anode total geometric surface area. The maximum operating current density (j_max_) was obtained from the curves of current density versus time. Every 2 days polarization curves were made as described by Sacco *et al*. [27].

### Electrochemical techniques

In the chronoamperometric assays, a 0.24 V was applied to the Toray paper WE. In CV assays, the potential was swept between -0.5 and 0.6 V at a scan rate of 1 mV/s. The first voltammogram is presented. All the potentials were measured against an Ag/AgCl_SAT_ reference electrode.

### Microscopic analysis

The *Dietzia* sp. RNV-4 biofilm images were obtained once the working electrodes were gently removed from every BES, in order to preserve the biofilm architecture and in sterile condition. After rinsing to eliminate excess dye, the samples were fixed to a glass slide. The confocal images were captured with a confocal laser scanning microscope (CLSM, Olympus FV 300, USA), using an argon laser (488 nm) as excitation source. The objective was 60X oil immersion, with zoom of 2.5X. Fluorescence was recorded with a low pass filter at 505 nm. A series of images were taken along the biofilm thickness (Z-axis) at regular intervals (1 μm), followed by a 3D volume reconstruction. XYZ images were processed using Image J software.

The electrodes were also observed with a scanning electron microscope (SEM, PHILIPS model XL30 TMP New Look) in vacuum conditions and the images were digitally registered by ANALYSIS software. The electrodes were treated and mounted as described by Sacco *et al*. [27].

## Results and Discussion

### Sequences and phylogenetic analysis RNV-4 isolated strain

Anoxic sediment from the shore zone at the Rio de la Plata River (Buenos Aires, Argentina) was used as starting material to assemble several 1 L sedimentary microbial fuel cells. Negative control SMFCs were performed (adding formaldehyde as a way to extinguish active microbial life, and also to avoid any further metabolic microbial activity (after, for example, spores germination and grow). In these controls the current production was minimal, so we can assume the presence of electroactive bacteria in the microbial communities of the sediments growing as a biofilm over the anode surface of the SMFC [27]. In order to isolate electroactive bacteria present in the microbial communities of the sediments growing as biofilm over the anode surface of the SMFC, different microbiological techniques were carried out (as described in Materials and Methods under the title “Microbiological techniques for the isolation and identification of the RNV-4 strain”).

As a result, different bacterial strains were isolated and one of these strains was selected based on its oxygen requirement, given our search of a new and versatile electrogenic facultative strain. The electroactive strains reported in literature up to this moment are facultative but use a few carbon sources as *S*. *oneidensis* or anaerobic as *Geobacter* [2–4]. This strain, first called RNV-4 (see next section), was the only one that grew both in aerobic and anaerobic conditions.

**Fig 2:**
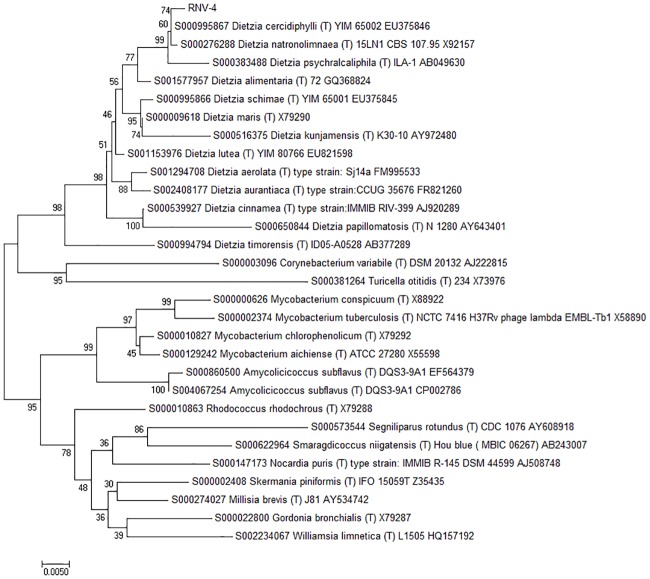
Phylogenetic tree of RNV-4 strain and closely related species based on 16S rRNA. The tree was constructed using the neighbor-joining method. The numbers at nodes indicate the percentages of occurrence of the branching order in 500 bootstrapped trees for values greater than 50%. Scale bar = 0.5% divergence.

To assess the identity of the RNV-4 strain, 16S rRNA regions were sequenced. The length of the sequence amplified was 814 of bp. The sequence in this study has been deposited in the GenBank database under accession number GenBank KY194791. BLAST was applied to find regions of similarity between biological sequences and the results showed that strain RNV-4 should indeed be assigned to the genus *Dietzia* and was most closely related to *Dietzia cercidiphylli* strain Y101 (99% sequence similarity), and *Dietzia natronolimnaea* strain 15LN1 (99% sequence similarity) and *Dietzia psychralcaliphila* ILA-1(99% sequence similarity). These three strains formed a distinct sub-cluster in the neighbor-joining, in which RNV-4 strain, *Dietzia cercidiphylli* strain Y101 and *Dietzia natronolimnaea* strain 15LN1 formed a distinct subline. For control, related species of the same family were used. These can be observed without groups in phylogenetic tree (Fig 2).

### Characterization of *Dietzia* sp. RNV-4 isolated strain

**Fig 3:**
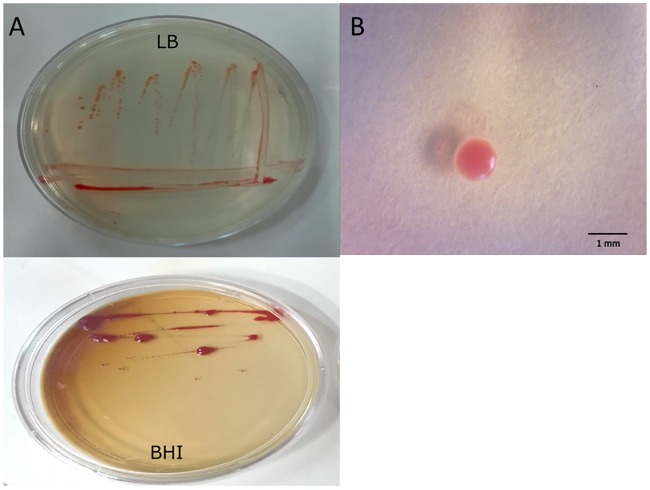
Morphology and colour of *Dietzia* sp. RNV-4. (A) Pigment expression after 48-h incubation at 32°C on Brain-Heart Infusion Agar (BHI) and Luria-Bertani agar (LB) media (B) Picture of a colony in LB agar obtained with a digital microscope (40X).

*Dietzia* sp. RNV-4 was characterized by their colony and cell morphology, motility, Gram and spore staining. This strain is a Gram-positive and non-spore forming coccus, which exhibit rod-shape curved in snapping division. Its colonies are circular, raised or convex, glistening from orange to coral red, with entire edges on solid media (BHI medium plus agar) (Fig 3).

The oxidase and catalase tests are part of the primary tests for bacteria characterization. These are rapid tests, which allow to know the tolerance of the bacteria to the oxygen. *Dietzia* sp. RNV-4 was tested positive for catalase, evidenced by bubbling, and negative for oxidase (absence of coloration in the treated filter paper) assays. Catalase-positive bacteria include strict aerobes as well as facultative anaerobes. They use oxygen as terminal electron acceptor. Generally, the cytochrome oxidase system is only found in aerobic organisms, some facultative anaerobes but strict anaerobes lack oxidase activity. The oxidase negative result just means that these organisms do not have the cytochrome c oxidase that oxidizes the test reagent. They may respire using other oxidases.

Catalase and oxidase assays were performed on all strains isolated (data not shown), so that potential candidates could be separated quickly (approx. 1 h). Then, growths were performed in the presence and absence of oxygen in culture plates and in liquid medium. Although these tests took longer (between 3 and 5 days), they served to reconfirm and characterize the growth of *Dietzia* sp. RNV-4.

Then, we cultured this strain in fluid thioglycollate and observed that after 72 h it grew at the top and all along the test tube, indicating that it grows at high and low concentrations of O_2_. Furthermore, assays of bacterial growth have been repeatedly done under controlled aerobic or anaerobic conditions and we observed bacterial growth in both tests; albeit at a slower growth rate in the anaerobic condition when compared to the aerobic cultures. In tests on solid culture, we observed the same result. According to the requirements of oxygen levels and the results obtained in the biochemical tests, *Dietzia* sp. RNV-4 could be described, for first time, as possibly facultative anaerobic bacteria.

An adhesion and biofilm forming ability of this strain was detected. We considered positive when an adherent layer of stained material was observed on the inner surface of the tube [26]. The OD_570_ values measured showed that *Dietzia* sp. RNV-4 formed biofilms on the glass surfaces, either in aerobic (1.01±0.04) or anaerobic (0.36±0.01) culture conditions. These results revealed that these bacteria possessed a high capacity for biofilm formation on glass surfaces. The indexes of bacteria adhering to surface and forming biofilms in the presence of O_2_ were approx. 3 times larger than those obtained in the absence of O_2_, probably related to the bacteria faster growth in aerobic conditions. Moreover, to our knowledge this was the first time that the ability of biofilm formation of *Dietzia* sp. RNV-4 either in aerobic or anaerobic conditions was described.

### Chronoamperometric analysis of biofilm-covered carbon paper electrodes

Although the mechanisms of substrate oxidation and electron transfer process related to electricity production in MFCs are of great importance to enhance the performance of a MFC, the nature of the underlying mechanisms is little known. For this reason, we construct a BES (Fig 1) in order to study an electrochemically active bacterial biofilm. In this way, the electron transfer mechanism as well as the molecules involved in the process can be described in detail.

**Fig 4:**
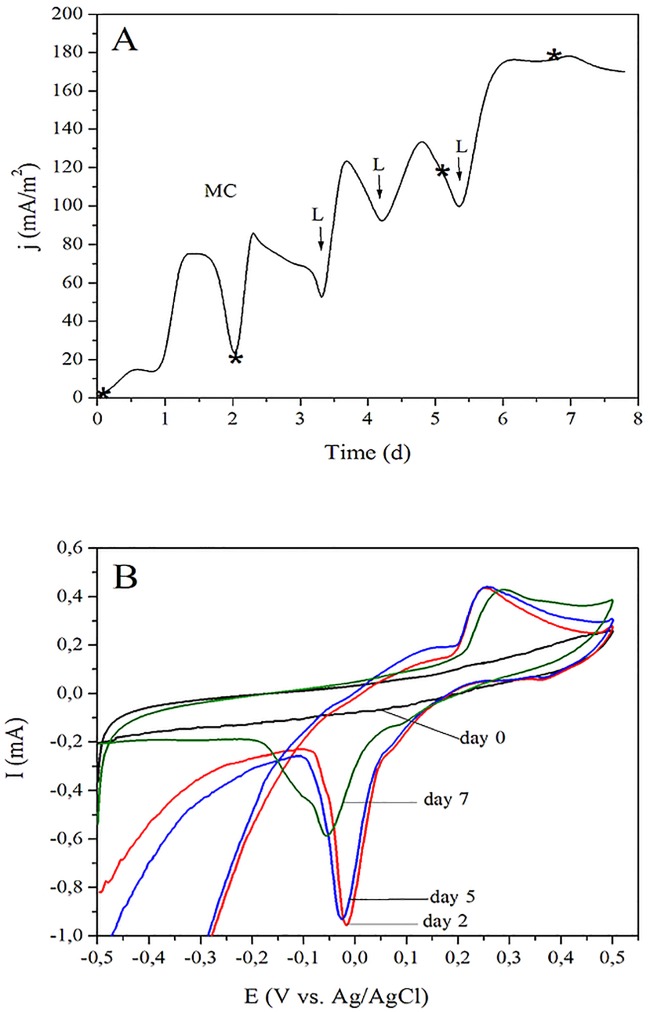
Electrochemical experiments performed with *Dietzia* sp. RNV-4, in the BES set-up. (A) Chronoamperometry after the inoculation. The medium change is indicated as MC and the addition of lactate as L (see Materials and Methods, section Assembly and operation of the BES). The star indicates the time when the CV experiments were done. (B) CVs at different stages of growth day 0 (just before inoculation, black), day 2 (red), day 5 (blue), and day 7 (green) of growth in the BES, indicated with star in A. Scan rate 1 mV/s.

Chronoamperometric assays were made in triplicate samples (n = 3) of *Dietzia* sp. RNV-4 cultured in a BES, to study the current production ability of the strain’s biofilm on Toray paper electrodes (Fig 4A). A current density of 16.2 ± 3.4 mA/m^2^ was observed 12 h after the BES inoculation with the strain. Current density reaches an apparent maximum of 76.1 ± 16.8 mA/m^2^ approximately 30 h after the beginning of the assay, limited probably by electron donor availability, which in turn limit not only bacterial metabolism but also growth. Current density begins to decrease at time equal to 36 h until it reaches a minimum value of 24.8 ± 5.5 mA/m^2^ at 48 h; then, the change of the spent culture medium was made. After the medium change and three consecutive lactate additions, achieving a final concentration of 20 mM, a maximum j of 176.6 ± 66.1 mA/m^2^ was reached at the end of the experiment (7 d) indicating that probably a mature biofilm was obtained (no further increase of j was observed).

As it can be seen in Fig 4A, the current increases after the addition of lactate in all the cases indicating that the lack of a carbon source would would limit the current production in this BES where *Dietzia* sp. RNV-4 is used as biological material.

A cyclic voltammetric analysis of the *Dietzia* sp. RNV-4 colonized electrode was performed, before the culture medium was changed or lactate was added, to verify if the biofilm produced active redox compounds (Fig 4B) [15,36,37]. We could see that two main peaks were found at voltammograms having biofilm; both, the anodic and cathodic peaks, increased their current when the biofilm matured. These increases are associated with the concentration of the active redox compounds, in the vicinity of the electrode.

Moreover, the position of the peaks can provide some identification information. The peak into a range of 0.288 and 0.254 V (vs. Ag/AgCl) matched closely with the values reported in the literature for canthaxanthin (0.309 V vs. Ag/AgCl) [38]. The difference between the value found by us and those reported in literature could be due to the different conditions used to perform the assays (electrode types, scan rates, medium composition and pH) [39]. It is known that some strains of *Dietzia* as *D*. *natronolimnaea* HS-1 produce carotenoids, being 90% of the carotenoids production specifically canthaxanthin (CTX) [40]. CTX molecule is known to play an active role in electron transfer processes in some photosynthetic systems.

To further validate the presence and accumulation of CTX in the medium, associated with the presence of the biofilm of *Dietzia* sp. RNV-4 on a carbon paper electrode, total carotenoids were extracted from these cell-free supernatants and absorbance spectroscopy measurements and CV were made (see supplementary information, SI). We used the criterion of the first derivative of absorbance vs. wavelength for the qualitative analysis of the peaks of absorbance spectra. We found that the commercial CTX extract was characterized by a single wide peak, with an absorbance maximum at 474 nm [41]. In the carotenoids extract from the BES, the maximum was at 470 nm, indicating that the principal carotenoid found in the supernatant is probably CTX or other closely related carotenoid (Figure A in S1 File). To confirm the identification of CTX, HPLC method was used, and the presence of CTX in the supernatants was demonstrated as the major component (Table 1). Additionally, CV was performed to the fraction extracted from the cell-free supernatant which would be confirming the presence of redox species related to CTX [40]. Furthermore, 1 mL aliquots of the commercial CTX extract (2 mg/L) were added sequentially to the cell-free supernatants. This was homogenized and then purged for 5 min with humidified sterile N_2_. We observed that the peak current increased as the concentration of CTX in the solution has increased by adding CTX (Figure B in S1 File). This suggests that CTX could be acting as a soluble redox mediator between the bacteria and the electrode surface.

**Table 1:** Carotenoids detected by HPLC method.

Sample	Number of peaks	Retention time (min)
Commercial canthaxanthin extract (CCE)	1	6.31
Cell-free supernatants extract	1	6.31–6.32
CCE + cell-free supernatants extract	1	6.31–6.32

The other peaks seen in the CVs (in the range of -0.05–0.0, see Fig 4B), which could then be associated with an active redox compound in particular, increased in a first step (for the first two days) and then began to decrease. This would indicate that the decrease in concentration occurs at the biofilm matured. The main peak observed can be related to the first EET mechanism previously proposed, which includes soluble electron shuttles, as flavins. It has been shown in some studies that this main mediators excreted by *Shewanella oneidensis* MR-1 are riboflavin and flavin mononucleotide [15]. However, we could not confirm the presence of flavins in our system.

To study the nature and localization of electroactive substances, CVs were performed in different fractions, including cell-free supernatants and bacterial suspensions from the centrifuged BES culture; in both cases, clean Toray paper electrodes were used. The obtained results were compared with the CVs of mature biofilms growing over the electrodes. The voltammograms of the cell-free supernatant (Figure C in S1 File) indicated that a mobile, suspended shuttle was present. The position of the anodic peak was very similar when compared with the one found in the biofilm (BES with *Dietzia* sp. RNV-4), therefore we can conclude that this soluble shuttle was also present in the mature biofilm. Although the chemical conditions at bulk and biofilm-covered electrode are probably different, the comparison between I_p_ (current peak position) at mature BES (Figure C in S1 File) and cell-free supernatant A showed a higher concentration of soluble mediators inside the biofilm and close to the electrode surface, when compared to the bulk concentrations.

The presented results would indicate that *Dietzia* sp. RNV-4, under these conditions, presents a mechanism of EET to solid electrodes, where a soluble electron shuttle would be involved.

**Fig 5:**
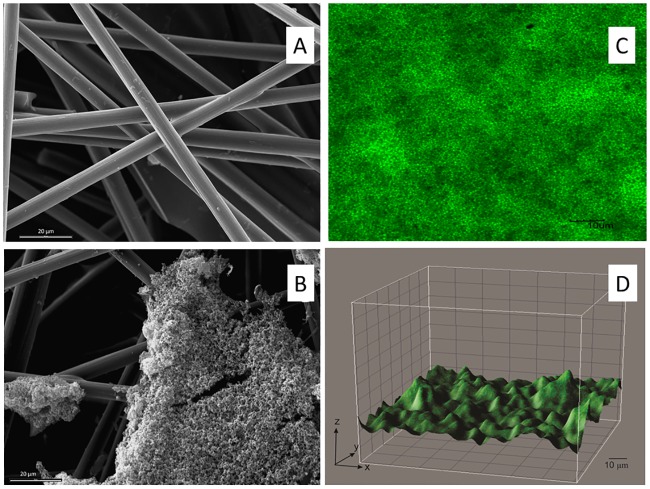
Microphotography of the working electrode used in the BES in order to understand the morphology of the biofilm formed by *Dietzia* sp. RNV-4 on carbon paper electrodes. (A) Electrode from a control experiment, 1000X, bar 20 μm, by SEM. (B) Biofilm of *Dietzia* sp. RNV-4, 1000X, bar 20 μm, by SEM. (C) Biofilm of *Dietzia* sp. RNV-4, 60X zoom 2.5, bar10 μm, by CLSM. (D) Biofilm of *Dietzia* sp. RNV-4, 60X zoom 2.5, by confocal microscopy. XYZ images were processed using the program Image J.

Higher superficial area and surface roughness of carbon electrodes (Toray paper) allows faster initial attachment and favor early biofilm formation. We studied the morphology of the *Dietzia* sp. RNV-4 biofilm on the electrodes by scanning electron microscope (SEM) and confocal microscope (Fig 5). In SEM images (Fig 5A and 5B) the development of the biofilm can be observed in the different regions of the electrode surface. It is possible to distinguish tri-dimensional agglomerates that emerge from the first layer. The biofilm shows a typical pillar like structure, with top fluffy layer and dense inner core; this is a typical structure, previously reported, for electroactive biofilms [17].

In the confocal microscopy images (Fig 5C), packed biofilms of *Dietzia* sp. RNV-4 could be seen on the electrode, showing also the typical structure for electroactive biofilms. This picture definitely revealed a biofilm between 12–18 μm of thickness.

### Current generation of *Dietzia* sp. RNV-4 in a MFC

MFC tests were conducted to evaluate the performance of the *Dietzia* sp. RNV-4 (MFC_D_) in energy production. The same test was made with *Shewanella oneidensis* MR-1 (MFC_S_), this allowed us to compare the results against one of the best known and characterized electrogenic bacteria, used as a control, in an identical set-up and conditions.

**Fig 6:**
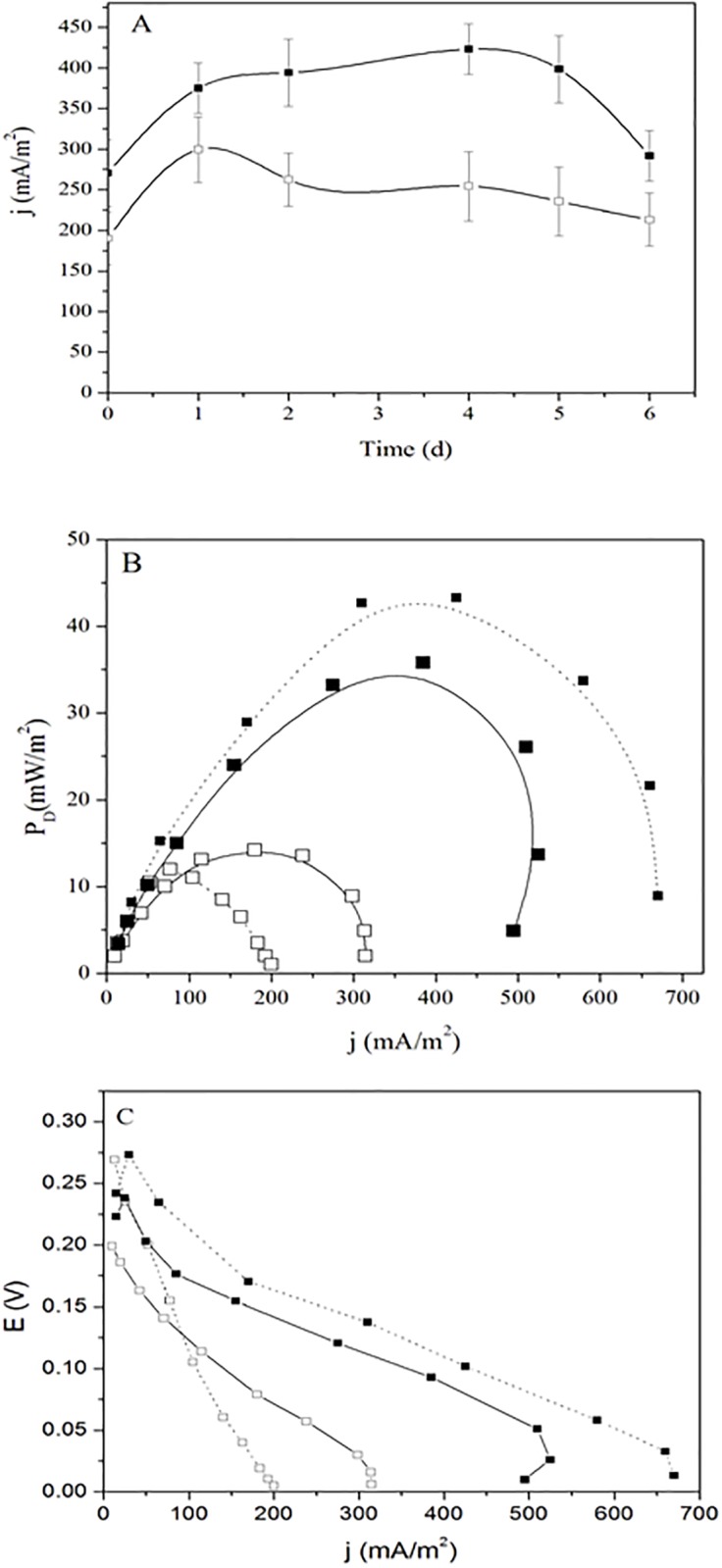
MFC experiments and bacterial strain comparison. (A) Current density vs. time measured from a MFC_S_ (*S*. *oneidensis* MR-1, closed squares) and from a MFC_D_ *(Dietzia* sp. RNV-4 (opened squares).n = 3, R_L_ = 4.68 kΩ. (B) Power density curves from the MFC_S_ (closed simbols squares) and MFC_D_ (opened squares) made after 1 (solid line) and 4 (dash line) days incubation. (C) Comparison of polarization curves from the MFC_S_ (closed squares) and MFC_D_ (opened squares) made after 1 (solid line) and 4 (dash line) days incubation.

The MFC_D_ and MFC_S_ produced a maximum current density (j_max_) of 299.4 ± 40.2 mA/m^2^ (n = 3) and of 431.3 ± 31.2 mA/m^2^ (n = 3), respectively. In both a fixed load resistor (R_L_) of 4.68 kΩ was used (Fig 6A). That shows that *S*. *oneidensis* produced about 40% more current that the *Dietzia* sp. RNV-4 based MFC, which could be adequate for energy production, considering the high diversity of carbon sources that *Dietzia* can use with respect to *Shewanella*.

The results obtained in this study suggested that *Dietzia* sp. RNV-4 could be an excellent candidate for the design and development of MFCs for power production and biosensors, given their ability to grow in aerobic or anaerobic conditions, an important advantage given that oxygen is toxic for several well-studied electrogenic bacterium. Further screening of the available electrogenic microbial diversity, and the strategies used for extracellular charge transfer to electrodes, are the basic information needed in the search for more efficient electrogenic organisms to be able to perform in present and future applications of bioelectrochemical systems. According to literature, *Dietzia* uses a variety of potential organic electron donors for current output such as lactate, pyruvate and amino acids [42]. That could withstand power output from biosensor using RNV-4. We could demonstrate that this strain grows in the presence of lactate as electron donor and fumarate as electron acceptor under aerobic conditions and lactate as sole carbon source in anaerobic conditions.

Several of the described species of *Dietzia* utilize aliphatic hydrocarbons as their sole carbon and energy source. There have also been reports on the degradation of aromatic compounds by *Dietzia* strains, including naphthalene, phenanthrene, benzoate, carbazole, quinoline, aniline and toluene [43,44,45]. So, it may have applications as a way to treat contaminated water whereas electricity is generated.

Canthaxanthin is a ketocarotenoid found in certain animals, plants, and microorganisms. Hence, because of its color and strong antioxidant activity, canthaxanthin is widely applied in the medical, pharmaceutical, cosmetic, poultry, fishery, and food industries [46,47]. At present, the large market for carotenoids is satisfied through chemical synthesis, although this has various disadvantages, as the chemical synthesis of carotenoids requires a very high level of control and can produce compounds that have undesired side effects and may be allergens in certain consumers. Therefore, in view of the global economic value of carotenoids and increased awareness of consumers, the production of these materials from natural sources has become an area of intensive investigation. With our MFC with *Dietzia* sp. RNV-4 we could, in addition to energy, produce biomass from which to extract the canthaxanthin.

### Polarization curves and power density curves

Power-current properties of MFC_D_ and MFC_S_ indicate the optimal current or voltage ranges at which each fuel cell can be operated to maximize the power density (P_D_) generation. With the MFC_D_ (n = 3) we obtain a P_D_ of 14.2 ± 2.1 mW/m^2^ (day 1), which then decreased to 12.0 ± 1.7 mW/m^2^ (day 4). The P_D_ measured with MFC_S_ was 35.8 ± 5.8 mW/m^2^, obtained at day 1 (Fig 6B); at day 4 the P_D_ was 43.3 ± 6.7 mW/m^2^. The power difference obtained with respect to *S*. *oneidensis* MR-1 was quite large, probably this is related to the use of a liquid basal medium (BM) for the experiments, that was needed in order to compare both strains.

The real potential of a real fuel usually below its equilibrium potential due to different types of losses. Three different types of losses can be distinguished as: ohmic, activation and concentration losses [48]. In order to compare the results of the different MFCs with *Dietzia* sp. RNV-4 or *S*. *oneidensis* MR-1, potential and polarization curves were made for both MFCs.

The polarization curve depicted in Fig 6C from MFCs with *Dietzia* sp. RNV-4 or *S*. *oneidensis* MR-1, as a function of j obtained during the stable phase of power generation (on the first and fourth day) presents E for resistances varying from 100 kΩ to 100 Ω.

The results obtained in first day with the MFC with *Dietzia* sp. RNV-4 show that the initial step of E decrease for low j depicted in Fig 6C suggested that had high activation losses [49,50]. For a j higher than 100 mA/m^2^ approx. the curve slope change and decreased, which indicated the prevalence of ohmic losses. After analyzing the value of each curve slope (linear regression) from the MFC with *Dietzia* sp. RN-4 and MFC with *S*. *oneidensis* MR-1, we saw that the slope was higher in the MFCD suggesting that activation and ohmic losses in this cell are higher. In the MFCs curve the slope decreased more smoothly than in the case of MFC_D_ for j higher than 100 mA/m^2^, which would indicate that ohmic losses are probably lower than in MFC with *Dietzia* sp. RNV-4. In both cases, MFCs with *Dietzia* sp. RNV-4 or *S*. *oneidensis* MR-1, the slope changes in the area of higher j show involve concentration losses.

Internal resistance, including anode, cathode, electrolyte, and membrane resistance, limits the power output of a MFC [48]. From the polarization curves, the internal resistance (R_int_) was estimated (slope of the linear portion, R_int_ = ΔE/Δj) for the different systems we assayed, (using the data shown in Fig 6C. The obtained values were probably related to the type of bacteria used; *Dietzia* sp. RNV-4 MFC shown a R_int_ of ca. 2701 Ω in the first day. However, on the fourth day increased to about 6613 Ω. So, the value of the internal resistance doubled. But when *S*. *oneidensis* MR-1 was assayed the value of the R_int_ was approx. 1600 Ω and remained approximately constant throughout the duration of the experiment. Similar values were obtained by Watson and Logan [51], using *S*. *oneidensis* MR-1 were the R_int_ was 1533 Ω. Since the internal resistance is affected by multiple variables of the MFC (electrode area, electrolyte ionic strength, pH, and others) [49]. Identification of the limiting factor of MFC requires the quantification of the contribution of each MFC component to R_int_.

## Conclusions

We report for the first time the electrochemical characterization of *Dietzia* sp.RNV-4, an isolated strain characterized physiologically as possibly facultative anaerobic bacteria, as a new electrogenic microorganism. The understanding of the several complementary charge transfer mechanism are important in the search for better microbial candidates for practical applications of MFCs. The results of the CVs suggest that *Dietzia* sp. RNV-4 canthaxanthin could be probably performing as the main soluble mediator, excreted or secreted by this bacterium; concentration of this mediator seem higher in the biofilm-electrode interface than in the bulk solution, perhaps by the adsorption of this molecule over Toray paper electrodes. The results obtained in this study suggest that *Dietzia* sp. RNV-4 could be an excellent candidate for the design and development of MFCs for power production and biosensors, given the ability to grow in aerobic or anaerobic conditions, an important advantage given that oxygen is toxic for several well-studied electrogenic bacterium. Further screening of the available electrogenic microbial diversity, and the strategies used for extracellular charge transfer to electrodes are the basic information needed in the search for more efficient electrogenic organisms, to be able to perform in present and future applications of bioelectrochemical systems. Complementary techniques as very sensitive immunological methods, coupled with fluorescence tags and confocal microscopy could be a necessary further step to understand direct electron transfer by macromolecular electron transporters associated to microbial membranes, as cytochromes are.

## Supporting Information

S1 File (**Figure A**) Absorption spectra of the ethanol fraction obtained from the supernatants, free of cells, of the BES. The full line represents commercial canthaxanthin extract and the dashed line represents the extract of the *Dietzia* sp. RNV-4 culture supernatant. **(Figure B)**. Response of anodic current peak of cell-free supernatant to the canthaxanthin aggregate. The data is not good enough to determined concentration of cell-free supernatant. **(Figure C).** Cyclic voltammetry at a scan rate of 1 mV sec^-1^ of BM medium at a clean electrode (a, black), *Dietzia* sp. RNV-4 in the BES system at 7 days (b, green), cell-free supernatant (c, orange) and pellet resuspended in BM (d, magenta).
(DOCX)

## References

[1] Abrevaya XC, Sacco NJ, Bonetto MC, Hilding-Ohlsson A, and Cortón E. Analytical applications of microbial fuel cells. Part I: Biochemical oxygen demand. Biosens. Bioelectron. 2015a;63:580–590. 24856922 10.1016/j.bios.2014.04.034

[2] Tender LM, Reimers CE, Stecher HA, Holmes DE, Bond DR, Lowy DA, Pilobello K, Fertig SJ, Lovley DR. Harnessing microbially generated power on the seafloor. Nat. Biotechnol. 2002; 20:821*–*825. 10.1038/nbt716 12091916

[3] Nevin KP, Richter H, Covalla SF, Johnson JP, Woodard TL, Orloff AL, Jia H, Zhang M, Lovley DR. Power output and columbic efficiencies from biofilms of *Geobacter sulfurreducens* comparable to mixed community microbial fuel cells. Environ. Microbiol. 2008; 10:2505–2514. 10.1111/j.1462-2920.2008.01675.x 18564184

[4] Lanthier M, Gregory KB, Lovley DR. Growth with high planktonic biomass in *Shewanella oneidensis* fuel cells. FEMS Microbiol.Lett.2008; 278: 29*–*35. 10.1111/j.1574-6968.2007.00964.x 17995953 PMC2228398

[5] Logan BE, and Rabaey K. Conversion of wastes into bioelectricity and chemicals by using microbial electrochemical technologies. Science. 2012; 337:686–90. 10.1126/science.1217412 22879507

[6] Kumar R, Singh L, and Zularisam AW. Exoelectrogens: Recent advances in molecular drivers involved in extracellular electron transfer and strategies used to improve it for microbial fuel cell applications. Renew. Sustainable Energy Rev. 2016; 56:1322–1336.

[7] Velasquez-Orta SB, Head IM, Curtis TP, Scott K, Lloyd JR, and von Canstein H. The effect of flavin electron shuttles in microbial fuel cells current production. Appl. Microbiol. Biotechnol. 2010; 85:1373–1381. 10.1007/s00253-009-2172-8 19697021

[8] von Canstein H, Ogawa J, Shimizu S, and Lloyd JR. Secretion of flavins by *Shewanella* species and their role as extracellular redox mediators. Appl. Environ. Microbiol. 2008; 74:615–623. 10.1128/AEM.01387-07 18065612 PMC2227709

[9] Marsili E, Baron DB, Shikhare ID, Coursolle D, Gralnick JA, and Bond DR. Shewanella secretes flavins that mediate extracellular electron transfer. PNAS. 2008a; 10:3968–3973. 10.1073/pnas.0710525105 PMC2268775 18316736

[10] Brutinel E, and Gralnick J. Shuttling happens: Soluble flavin mediators of extracellular electron transfer in *Shewanella*. Appl. Microbiol. Biotechnol. 2012; 93:41–48. 10.1007/s00253-011-3653-0 22072194

[11] Kotloski NJ, and Gralnick JA. Flavin electron shuttles dominate extracellular electron transfer by Shewanella oneidensis. mBio. 2013; 4(1):e00553–12. 10.1128/mBio.00553-12 23322638 PMC3551548

[12] Liu Y, Wang Z, Liu J, Levar C, Edwards MJ, Babauta JT, et al A trans-outer membrane porin-cytochrome protein complex for extracellular electron transfer by *Geobacter sulfurreducens* PCA. Environ. Microbiol. Rep. 2014; 6:776–85. 10.1111/1758-2229.12204 25139405 PMC4282303

[13] Reguera G, McCarthy KD, Mehta T, Nicoll JS, Tuominen MT, and Lovley DR. Extracellular electron transfer via microbial nanowires. Nature. 2005; 435:1098–110. 10.1038/nature03661 15973408

[14] Malvankar NS, Yalcin SE, Tuominen MT, and Lovley DR. Visualization of charge propagation along individual pili proteins using ambient electrostatic force microscopy. Nat. Nanotechnol. 2014; 9:1012–1017. 10.1038/nnano.2014.236 25326694

[15] Marsili E, Rollefson JB, Baron D, Hozalski RM, and Bond DR. Microbial biofilm voltammetry: Direct electrochemical characterization of catalytic electrode-attached biofilms. Appl. Environ. Microbiol. 2008b; 74:7329–7337. 18849456 10.1128/AEM.00177-08 PMC2592900

[16] Inoue K, Leang C, Franks AE, Woodard TL, Nevin KP, and Lovley DR. Specific localization of the *c*-type cytochrome OmcZ at the anode surface in current-producing biofilms of *Geobacter sulfurreducens*. Environ. Microbiol. Rep. 2011; 3:211–217. 10.1111/j.1758-2229.2010.00210.x 23761253

[17] Jain A, Zhang X, Pastorella G, Connolly JO, Barry N, Woolley R, Krishnamurthy S, et al Electron transfer mechanism in *Shewanella loihica* PV-4 biofilms formed at graphite electrode. Bioelectrochemistry. 2012; 87:28–32. 10.1016/j.bioelechem.2011.12.012 22281091

[18] Pirbadian S, Barchinger SE, Leung KM, Byun HS, Jangir Y, Bouhenni RA, et al Shewanella oneidensis MR-1 nanowires are outer membrane and periplasmic extensions of the extracellular electron transport components. PNAS. 2014; 11:12883–12888. 10.1073/pnas.1410551111 PMC4156777 25143589

[19] Wang VB, Kirchhofer ND, Chen X, Tan MYL, Sivakumar K, Cao B et al Comparison of flavins and a conjugated oligoelectrolyte in stimulating extracellular electron transport from *Shewanella oneidensis* MR-1. Electrochem. Commun. 2014; 41:55–58.

[20] Baron DB, LaBelle E, Coursoll D, Gralnick JA, and Bond DR. Electrochemical measurements of electron transfer kinetics by *Shewanella oneidensis* MR-1. J. Biol. Chem. 2009; 284:28865–28873. 10.1074/jbc.M109.043455 19661057 PMC2781432

[21] Snider RM, Strycharz-Glaven SM, Tsoi SD, Erickson JS, and Tender LM. Long-range electron transport in *Geobacter sulfurreducens* biofilms is redox gradient driven. Proc. Natl. Acad. Sci. USA. 2012; 109:15467–15472. 10.1073/pnas.1209829109 22955881 PMC3458377

[22] Guo K, Freguia S, Dennis PG, Chen X, Donose BC, Keller J, et al Effects of surface charge and hydrophobicity on anodic biofilm formation, community composition,and current generation in bioelectrochemical systems. Environ. Sci. Technol. 2013; 47:7563–70. 10.1021/es400901u 23745742

[23] Jayashree C, Tamilarasan K, Rajkumar M, Arulazhagan P, Yogalakshmi KN, Srikanth M, et al Treatment of seafood processing wastewater using upflow microbial fuel cell for power generation and identification of bacterial community in anodic biofilm. J. Environ. Manage. 2016; 180:351–358. 10.1016/j.jenvman.2016.05.050 27254294

[24] Samsudeen N, Radhakrishnan TK, and Matheswaran M. Bioelectricity production from microbial fuel cell using mixed bacterial culture isolated from distillery wastewater. Bioresour. Technol. 2015; 195: 242–247. 10.1016/j.biortech.2015.07.023 26212679

[25] Abrevaya XC, Sacco NJ, Bonetto MC, Hilding-Ohlsson A and Cortón E. Analytical applications of microbial fuel cells. Part II: Toxicity, microbial activity and quantification, single analyte detection and other uses. Biosens. Bioelectron. 2015b; 63:591–601. 24906984 10.1016/j.bios.2014.04.053

[26] Kumar R, Lakhveer S, Wahid ZA, and Din MF. Exoelectrogens in microbial fuel cells toward bioelectricity generation: A review. Int. J. Energy Res. 2015; 39:1048–1067.

[27] Sacco NJ, Figuerola ELM, Pataccini G, Bonetto MC, Erijman L, and Cortón E. Performance of planar and cylindrical carbon electrodes at sedimentary microbial fuel cells. Bioresour. Technol. 2012; 126:328–335. 10.1016/j.biortech.2012.09.060 23142927

[28] Hungate RE. A roll tube method for cultivation of strict anaerobes In: Methods in Microbiology. Norris JR, Ribbons EW (eds). New York, Academic Press, 1969 pp. 117–132.

[29] Kumar S, Stecher G, and Tamura K. MEGA7: Molecular Evolutionary Genetics Analysis version 7.0 for bigger datasets. Molecular Biology and Evolution. 2016; 33:1870–1874. 10.1093/molbev/msw054 27004904 PMC8210823

[30] Merchant IA, Packer RA. Veterinary Bacteriology and Virology. 7th Ed; The Iowa State University Press, Ames, Iowa, US 1969

[31] Schaeffer AB, Fulton M, A simplified method of staining endospores. Science. 1933; 77, 194 10.1126/science.77.1990.194 17741261

[32] Christensen GD, Simpson W.A, Younger JJ, Baddour LM, Barrett FF, Melton DM, and Beachey EH. Adherence of coagulase-negative staphylococci to plastic tissue culture plates: A quantitative model for the adherence of staphylococci to medical devices. J. Clin. Microbiol. 1985; 22:996–1006. 3905855 10.1128/jcm.22.6.996-1006.1985 PMC271866

[33] Mahon CR, Lehman DC, and Manuselis G. Textbook of Diagnostic Microbiology, fourth ed, W.B. Saunders Co., Philadelphia 2011.

[34] Gaby WL, and Hadley C. Practical laboratory test for the identification of *Pseudomonas aeruginosa*. J. Bacteriol. 1957; 74:356–358. 13475249 10.1128/jb.74.3.356-358.1957 PMC314647

[35] Esfahani-Mashhour M, Moravej H, Mehrabani-Yeganeh H, and Razavi SH. Evaluation of coloring potential of *Dietzia natronolimnaea* biomass as source of canthaxanthin for egg yolk pigmentation. Asian-Aust. J. Anim. Sci. 2009; 22:254–259.

[36] LaBelle E, and Bond DR. Bio-electrochemical systems: From extracellular electron transfer to biotechnological application. Integrated Environmental Technology Series. Wageningen University, The Netherlands. 2009.

[37] Harnisch F, and Freguia S. A basic tutorial on cyclic voltammetry for the investigation of electroactive microbial biofilms. Chem. Asian J. 2012; 7:466–475. 10.1002/asia.201100740 22279004

[38] Liu D, Gao Y, and Kispert LD. Electrochemical properties of natural carotenoids. J. Electroanal. Chem. 2000; 488:140–150.

[39] Bard AJ and Faulkner LR. Electrochemical Methods: Fundamentals and Applications, 2nd ed, Wiley, New York, 2001.

[40] Khodaiyan F, Razavi SH, Emam-Djomeh Z, Mousavi SMA, and Hejazi MA. Effect of culture conditions on canthaxanthin production by *Dietzia natronolimnaea* HS-1. J. Microbiol. Biotechnol. 2007; 17:195–201. 18051749

[41] Asker D, and Ohta Y. Production of canthaxanthin by extremely halophilic bacteria. J. Biosci. Bioeng. 1999;88:617–621. 16232673 10.1016/s1389-1723(00)87089-9

[42] Yumoto I, Nakamura A, Iwata H, Kojima K, Kusumoto K, Nodasaka Y, Matsuyama H. *Dietzia psychralcaliphila* sp. nov., a novel facultatively sychrophilic alkaliphile that grows on hydrocarbons. Int. J. Syst. Evol. Microbiol. 2002; 52:85–90 10.1099/00207713-52-1-85 11837320

[43] Bødtker G, Hvidsten IV, Barth T, Torsvik T. Hydrocarbon degradation by Dietzia sp. A14101 isolated from an oil reservoir model column. Anton. Leeuw. Int. J. G. 2009; 96:459–469. 10.1007/s10482-009-9359-y 19565350

[44] von der Weid I, Marques JM, Cunha D, Lippi RK, dos Santos SCC, Rosado AS, Lins U, Seldin L. Identification and biodegradation potential of a novel strain of *Dietzia cinnamea* isolated from a petroleum-contaminated tropical soil. Syst. Appl. Microbiol. 2007; 30: 331–339. 10.1016/j.syapm.2006.11.001 17174505

[45] Bhosale P. and Bernstein P.S. Microbial xanthophylls. Appl.Microbiol.Biotechnol. 2005; 68: 445–455. 10.1007/s00253-005-0032-8 16001255

[46] Edge R, McGarvey D. and Truscott T. Carotenoids as antioxidants—a review. J. Photochem. Photobiol. B Biol. 1997; 41: 189–200. 10.1016/s1011-1344(97)00092-4 9447718

[47] Nelis JH and De Leenheer PA. Microbial sources of carotenoid pigments used in foods and feeds. J. Appl. Bacteriol. 1991; 70:181–191.

[48] Logan B.E., Power Generation, In Microbial Fuel Cells. John Wiley &Sons, New York 2008 pp. 44–60

[49] Logan BE, Hamelers B, Rozendal R, Schröder U, Keller J, Freguia S, Aelterman P, Verstraete W, Rabaey K. Microbial fuel cells: methodology and technology. Environ. Sci. Technol. 2006; 40:5181–5192. 16999087 10.1021/es0605016

[50] Lovley DR. Bug juice: harvesting electricity with microorganisms. Nat. Rev. Microbiol. 2006; 4: 497–508. 10.1038/nrmicro1442 16778836

[51] Watson VJ and Logan BE. Power production in MFCs inoculated with *Shewanella oneidensis* MR-1 or mixed cultures. Biotechnol. Bioeng. 2010; 105:489–498. 10.1002/bit.22556 19787640

