# Supplementary material for: Isolation and Characterization of a Novel Electrogenic Bacterium, Dietzia sp. RNV-4
Source: PLoS One. 2017 Feb 13;12(2):e0169955. doi: 10.1371/journal.pone.0169955 (PMC5305051; doi:10.1371/journal.pone.0169955)
Supplement: S1 File — (Figure A) Absorption spectra of the ethanol fraction obtained from the supernatants, free of cells, of the BES. The full line represents commercial canthaxanthin extract and the dashed line represents the extract of the Dietzia sp. RNV-4 culture supernatant. (Figure B). Response of anodic current peak of cell-free supernatant to the canthaxanthin aggregate. The data is not good enough to determined concentration of cell-free supernatant. (Figure C). Cyclic voltammetry at a scan rate of 1 mV sec-1 of BM medium at a clean electrode (a, black), Dietzia sp. RNV-4 in the BES system at 7 days (b, green), cell-free supernatant (c, orange) and pellet resuspended in BM (d, magenta). (DOCX) [file pone.0169955.s001.docx]

***Supplementary information***

Isolation and Characterization of a Novel Electrogenic Bacterium, *Dietzia* sp. RNV-4

Natalia J. Sacco^*^, María C. Bonetto, Eduardo Cortón

Laboratory of Biosensors and Bioanalysis (LABB), Departamento de Química Biológica and IQUIBICEN-CONICET, Facultad de Ciencias Exactas y Naturales, Universidad de Buenos Aires, Ciudad Universitaria. Ciudad Autónoma de Buenos Aires, Buenos Aires, Argentina.

*Correspondence Author

E-mail: nsacco@qb.fcen.uba.ar (NS)

***Validation of the presence and accumulation of Canthaxanthin***

To further validate the presence and accumulation of canthaxanthin (CTX) in the medium, associated with the presence of the biofilm of *Dietzia* sp*.* RNV-4 on a carbon paper electrode, total carotenoids were extracted from these cell-free supernatants and absorbance spectroscopy measurements was made. We used the criterion of the first derivative of absorbance vs. wavelength for the qualitative analysis of the peaks of absorbance spectra. We found that the commercial CTX extract was characterized by a single wide peak, with an absorbance maximum at 474 nm (Figure A).


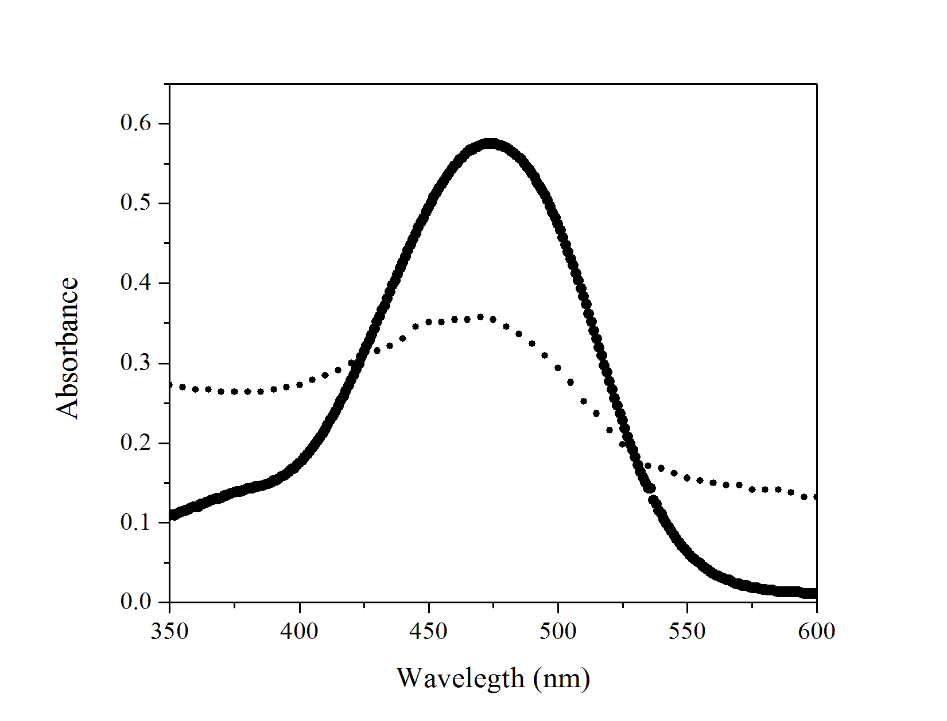


**Figure A.**

To confirm the identification of CTX, CV was performed to the fraction extracted from the cell-free supernatant which would be confirming the presence of redox species related to CTX. Furthermore, 1 mL aliquots of the commercial CTX extract (2 mg/L) were added sequentially to the cell-free supernatants. This was homogenized and then purged for 5 min with humidified sterile N_2_. We observed that the peak current increased as the concentration of CTX in the solution has increased by adding CTX (Figure B), the initial volume was 40 mL, and initial I_p_ was 0.112 mA.

**Figure B.**

***Characterization of electron transfer mechanisms***

Pure bacterial cultures can differ in the way that electrons are transferred to the electrode; either soluble or membrane-bound components can be accountable of the process. To study the nature and localization of electroactive substances, CVs were performed in different fractions, including cell-free supernatants and bacterial suspension from the centrifuged BES culture; in both cases, clean Toray paper electrodes were used; the obtained results were compared with the CVs of mature biofilm growing over the electrodes. As a control, a CV of the BM medium with a clean carbon electrode was made, as expected, no peaks are observed (Figure C, voltammogram a). The voltammogram of *Dietzia* sp. RNV-4 in the BES at day 7 (Figure C, voltammogram b) had an anodic peak at approx. 0.288 V (vs. Ag/AgCl) and three cathodic peaks with E_pc_ ~ -0.055 (main peak) and two small peaks, one is only notable as a shoulder with E_p_ ~ -0.060 V and other with E_p_ ~ 0.085 V (all vs. Ag/AgCl). The E_p_ of the small peaks was obtained by derivation of the original data.

The voltammogram for cell-free supernatant (Figure C, voltammogram c) had two peaks, with E_pa_ ~ 0.267 V and E_pc_ ~ 0.040 V (vs. Ag/AgCl), whereas the bacterial suspension (Figure C, voltammogram d) CV produced less notable peaks, with E_pa_ ~ 0.184 V and E_pc_ ~ -0.124 V (vs. Ag/AgCl). The voltammograms of the cell-free supernatant indicated that a mobile, suspended shuttle was present; the position of the anodic peak was very similar when compared with the one found in the biofilm (BES with *Dietzia* sp*.* RNV-4), therefore we can conclude that this soluble shuttle was also present in the mature biofilm; one cathodic peak was also present in both voltammograms, even though displaced to more negative potentials. I_p_ in a voltammogram is linearly proportional to the electroactive substances; although the chemical condition at bulk and biofilm-covered electrode are probably different, the comparison between I_p_ (E_p_ ~ 0.270 V vs. Ag/AgCl), at mature BES (Figure C, voltammogram b) and cell-free supernatant (Figure C, voltammogram c) showed an I_p_ of 0.26 and 0.11 mA, respectively, showing a higher concentration of soluble mediators inside the biofilm and close to the electrode surface, when compared with the bulk concentrations.

Remarkably, the washed bacterial suspension anodic peak (E_pc_ ~ -124) could be related to the shoulder (E_pc_ ~ -0.060 V) present in BES voltammogram (Figure C, voltammogram d). Less evident, the cathodic peaks had also a close match between both voltammagrams (washed bacterial suspension, E_p_ ~ 0.184 V, compared with E_p_ ~ 0.267 V), all expressed vs. Ag/AgCl. The peaks presented in the washed bacterial suspension are probably associated to relatively non-mobile, membrane associated charge transporters, as cytochromes are; by comparison with BES at Figure C and Fig 4 B, seems to be related to the two new small peaks that appeared at day 7 BES, but they were not present before (E_p_ ~ -0.060 V and E_p_ ~ 0.085 V vs. Ag/AgCl). Other authors have associated peaks with E_p_ ~ -0.070 V vs. Ag/AgCl to type C cytochromes present in the external membrane of *Shewanella loihica* (Logan, 2009), but we have not enough information about the nature of the non-mobile charge transporters that we found.

The presented results would be indicating that *Dietzia* sp. RNV-4, under these conditions, presents a mixed mechanism of EET to solid electrodes, where a soluble electron shuttle and a membrane-associated shuttle would be involved, being the membrane-associated shuttle linked with mature biofilm.


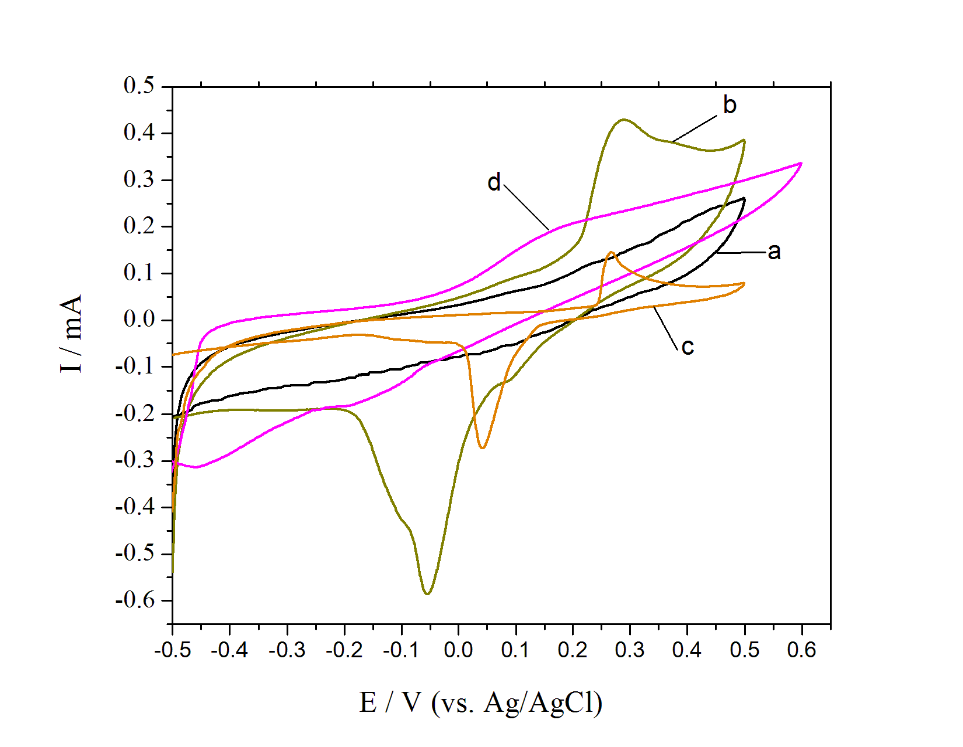


**Figure C.**

**Reference**

Logan BE. Exoelectrogenic bacteria that power microbial fuel cells. Nat. Rev. Microbiol. 2009; 7: 375-381.
